# Newly Developed System for Acetamiprid Residue Screening in the Lettuce Samples Based on a Bioelectric Cell Biosensor

**DOI:** 10.3390/bios10020008

**Published:** 2020-01-24

**Authors:** Theofylaktos Apostolou, Konstantinos Loizou, Agni Hadjilouka, Antonios Inglezakis, Spyridon Kintzios

**Affiliations:** 1 EMBIO Diagnostics Ltd., Athalassas Ave 8, Strovolos, 2018 Nicosia, Cyprus; k.loizou@embiodiagnostics.eu (K.L.); agni@embiodiagnostics.eu (A.H.); a.inglezakis@embiodiagnostics.eu (A.I.); 2 Laboratory of Cell Technology, Department of Biotechnology, Agricultural University of Athens, 75 Iera Odos St., 11855 Athens, Greece; skin@aua.gr

**Keywords:** cell based biosensor, acetamiprid, bioelectric recognition assay, membrane-engineering

## Abstract

Population growth and increased production demands on fruit and vegetables have driven agricultural production to new heights. Nevertheless, agriculture remains one of the least optimized industries, with laboratory tests that take days to provide a clear result on the chemical level of produce. To address this problem, we developed a tailor-made solution for the industry that can allow multiple field tests on key pesticides, based on a bioelectric cell biosensor and the measurement of the cell membrane potential changes, according to the principle of the Bioelectric Recognition Assay (BERA). We developed a fully functional system that operates using a newly developed hardware for multiple data sources and an Android application to provide results within 3 min. The presence of acetamiprid residues caused a cell membrane hyperpolarization, which was distinguishable from the control samples. A database that classified samples Below or Above Maximum Residue Levels (MRL) was then created, based on a newly developed algorithm. Additionally, lettuce samples were analyzed with the conventional and the newly developed method, in parallel, revealing a high correlation on sample classification. Thus, it was demonstrated that the novel biosensor system could be used in the food supply chain to increase the number of tested products before they reach the market.

## 1. Introduction

Over the last years, significant concerns about public health, environmental quality, as well as food safety have been on the rise, due to the large quantities of pesticides used in production on a daily basis [1]. It is well-known that pesticides are potentially toxic to humans and that the level of risk is directly related to the rate of daily intake of chemically contaminated food [2]. The quantity of a pesticide that remains in or on food is called pesticide residue. Detection of pesticide residues is a major concern of food safety experts and requires a rapid method of pesticide residue detection so that people are not exposed to potential health risks. The Environmental Protection Agency (EPA) has set the maximum levels of pesticide residues on particular food, however, detecting pesticides at these levels remains a challenge [3].

Acetamiprid belongs to the new neonicotinoid class of systemic broad-spectrum insecticides and is widely used in modern agriculture as a replacement insecticide of organophosphorus and other conventional insecticides to control sucking-type insects on various crops, especially on leafy vegetables, fruits, and tea trees [4,5]. Use of acetamiprid is banned in many countries, because of the severe environmental pollution and pesticide-resistance [6]. The excessive and improper usages have caused heavy pesticide residues in grains, agricultural products or food.

Acetamiprid can generate potential health risk to human beings since it can affect human peripheral blood lymphocytes and lead to DNA damage [7,8]. Due to these risks on human health and the harmful effects on the environment, it is necessary to develop analytical methods for sensitive and rapid detection of acetamiprid in food to keep people from potential health risks.

Various assay methods are available for the analysis of acetamiprid residues in fresh products, but most laboratories extract results using high-performance liquid chromatography (HPLC) [9], gas chromatography (GC) [10], flow and antibody-based immunoassays (AIA) [11], mass spectrometry (MS) [12] and enzyme-linked immunosorbent assays (ELISAs) [13]. However, these methods have high equipment costs, are time-consuming and require special training. Thus, they cannot keep up with the required volumes of produce and field-testing [14]. Therefore, it is necessary to develop rapid, simple, cost-effective, sensitive and portable alternatives for the determination of acetamiprid in order to reduce the risk to public health.

Biosensors as a fast, cost-effective, portable and sensitive tool, are a promising alternative method for testing food contamination. Biosensors’ mode of action is based on their ability to detect the target and transform this recognition into a detectable signal [15,16]. Depending on the final form of the detectable signal, the biosensors are divided into optical, piezoelectric, mechanical, and electrochemical. Electrochemical biosensors are the most frequently used to date in pesticide detection [17,18]. At the same time, because of their high sensitivity, low cost of manufacture and their size, they are excellent candidates for the development of portable biosensors [19,20]. The biological elements commonly used in target detection are antibodies, enzymes, and even whole cells. Immunosensors, unlike enzyme biosensors that evaluate total toxicity, have the ability to be specific for a molecule. This is achieved due to the high affinity of the antibodies (Ab) or antigens (Ag) immobilized on the surface of a transducer and the target analytes that are Ag or Ab, respectively [21,22].

Live cell-based biosensors have been proven to have high selectivity, sensitivity, and rapid response times. These detection systems, such as the Bioelectric Recognition Assay (BERA), have already been used in a wide range of applications with a focus on environmental, chemical, and medical applications [23]. To date, a major application of the BERA method is its use as a pesticide residues screening tool [24,25]. Despite the ease of use of the system by an experienced user, the disadvantage of examining the data resulting from the biosensor response and the empirical way of deriving the result, remains a challenge.

The most significant problems nowadays are the lack of true portable pesticide screening tools that can screen the food production on processing sites and the extremely low number of samples tested annually by the existing methods. A portable, fast, and smart biosensor device could solve these issues and provide a quick control solution to international food producers and industry. Due to biosensors’ rapid and high throughput pesticide residue testing capability, this method can be an accurate and indispensable tool that will provide food companies and growers with the ability to control the presence of residues regularly, hence preventing potential risks. 

In the present study, we report the development of a new version of a portable BERA-type sensor for field application based on mammalian cells, which have been “membrane-engineered” with an anti-acetamiprid monoclonal antibody. The method is based on the electro insertion of specific antibodies on the surface of cultured mammalian cells, at high density. Rendering the cell makes it a very specific responder against homologous binding to the membrane-bound antibodies according to the principles of the molecular identification through membrane-engineering [26,27]. The novel portable sensor is combined with a sophisticated algorithm embedded in a user-friendly software, obtaining an automated result in a smartphone, without the need of examining the biosensor response data by the user.

## 2. Materials and Methods

### 2.1. Materials

Monkey African green kidney (Vero) cells cultures were originally provided from LGC Promochem (Teddington, UK). Acetamiprid was purchased from Sigma-Aldrich (Taufkirchen, Germany). Monoclonal antibodies against acetamiprid were obtained from Creative Diagnostics (Upton, New York). The experimental procedure was performed according to the European Method EN 15662. QuEChERS (Quick, Easy, Cheap, Effective, Rugged, and Safe) extraction was performed using the Bond Elut QuEChERS P/N 5982 extraction kit containing 4 g MgSO_4_, 1 g NaCl, 1 g NaCitrate, 0.5 g disodium citrate sesquihydrate. A dispersive solid-phase extraction salt and sorbent kit were tested; Bond Elut QuEChERS P/N 5982-5221 containing 25 mg PSA 2.5 mg GCB 150 mg MgSO_4_. Bond Elut QuEChERS were purchased from Agilent (Agilent Technologies, Lake Forest, CA, USA). All other reagents were purchased from Sigma-Aldrich (Taufkirchen, Germany).

### 2.2. Cell Culture and Sensor Fabrication from Vero Cells

Vero cells were cultured using Dulbecco’s medium with 10% fetal bovine serum (FBS), 10% antibiotics (streptomycin-penicillin) and 10% l-glutamine and l-alanine. Following, cell were detached from the culture vessel with the use of trypsin/EDTA for 10 min at 37 ℃. Afterward, cells concentrated by centrifugation (2 min, 1200 rpm, 25 ℃), at a density of 2.5 × 10^6^ mL^−1^.

Biosensors used were membrane-engineered cells. In order to make them selective, the protocol requires the insertion of acetamiprid monoclonal antibodies into the membrane of Vero cells, following a modified protocol of Zeira et al. [28]. The following step on the fabrication process is the centrifugation at 1000 rpm for 6 min and then resuspended in 40 μL of Dulbecco’s medium (10% fetal bovine serum-FBS). Afterward, the modified cells were incubated with the antibodies (0.4 μg mL^−1^) on ice. After 20 min the mixture was transferred to appropriate electroporator cuvettes. Electroinsertion was performed by applying two square electric pulses at 1800 V/cm. Following electroinsertion, the mixture was transferred in small Petri dishes (60 × 15 mm) which contained 3 mL of Dulbecco’s medium provided with 10% fetal bovine serum (FBS) and incubated at 37 ℃ and 5% CO_2_ for 1 d. Finally, before the measurement of the samples, the medium was removed from the Petri dishes and cell detachment from the culture was achieved by adding 2 mL of nutrient medium and collecting the cells in Eppendorf tubes.

### 2.3. Sample Preparation

In order to assure the correct analogy of the lettuce sample, 10g per lettuce was placed on a 50 mL centrifuge tube. Afterward 10mL of Acetonitrile was added on the tube, QuEChERS [29] extraction salt packet (EN method: 4 g MgSO_4_, 1 g NaCl, 1 g NaCitrate, 0.5 g disodium citrate sesquihydrate) and by a ceramic homogenizer. The tube was instantly sealed and shaken by hand for 60 s. Afterward the tube was centrifuged for 5 min at 3000 U/min. The top acetonitrile layer was collected, and aliquots were taken for subsequent clean-up. The dispersive solid-phase extraction salt and sorbent kit Bond Elut QuEChERS P/N 5982-5221 containing 25 mg PSA 2.5 mg GCB 150 mg MgSO_4_ was used. Following the clean-up process, was done using a 1 mL aliquot of each extract was added to the dSPE tubes. The tubes were gently shaken for 2 min and then centrifuged for 5 min at 3000 U/min centrifuge. Finally, a 0.5 mL portion of the supernatant extract was removed and placed into a glass centrifuge tube. The supernatant was evaporated to dryness under a stream of nitrogen and the residue was re-dissolved in 10% DMSO, thus limiting possible toxic effects of the solvent on the cellular biorecognition elements

### 2.4. Spiking Lettuce Extract Samples

Stock solution of 50 μg mL^−1^ acetamiprid was prepared in 10% DMSO. For the preparation of the final concentrations, dilution series were performed by using acetamiprid-free samples. The final concentrations used to build the database were: 15, 10, 8.75, 7.5, 6.25, 5, 4.5, 4, 3.5, 3, 2.5, 2, 1.5, and 0.5 μg mL^−1^. Acetamiprid-free samples were also used as controls.

### 2.5. Assay Procedure

#### 2.5.1. Biosensor Device

**Figure 1:**
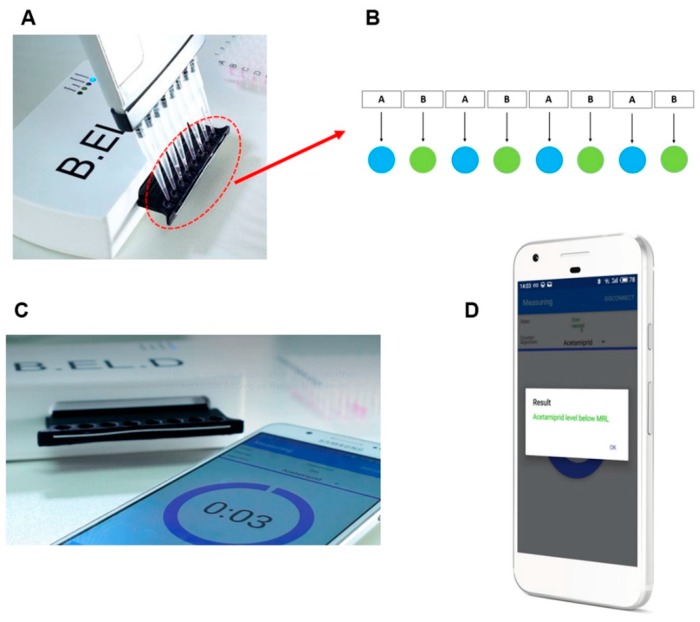
(**A**) Use of multichannel automated pipette for the addition of (i) 36 μl of cells and then (ii) 4 μl of samples on the electrode surface. (**B**) Arrangement of cells on the electrode surface (A: non-engineered cells, B: engineered cells). (**C**) Device connected with smartphone and measurement start. (**D**) The result appears on the smartphone screen.

In order to achieve the detection requirements of the system, a customized hardware portable device was developed (EMBIO DIAGNOSTICS Ltd, Nicosia, Cyprus). The device is using high accuracy A/D converters to measure electric signals from the cellular biorecognition elements, allowing for high throughput screening, multichannel, parallel measurements, wireless broadcasting and high speed of assay. The portable device is a multichannel potentiometer, having a replaceable connector of eight channels of SPE electrodes connecting on the underside (Figure 1C) and operating according to the principle of the Bioelectric Recognition Assay. The system is based on a modification of a design previously reported by Apostolou et al. [30]. The system is able to connect via Bluetooth 4.0 with a smartphone (Figure 1C) and provide changes of the electric properties of cells from up to eight simultaneous measurements from respective eight carbon screen-printed electrodes (working electrode: carbon, reference: Ag/AgCl) on a disposable sensor strip (Gwent, UK) (Figure 1C). The counter electrode was cancelled out by the measuring system. 

Biosensor cells, with or without antibody, were added first on the top of each of the eight carbon screen-printed electrodes contained in each disposable sensor strip (36 μL ≈ 50 × 10^3^ cells), following the arrangement shown in Figure 1B. Next, with the help of a multichannel automatic pipette, 4 μL of sample (either standard solution of the pesticide either lettuce extract) were added (Figure 1A). Immediately after the addition of the sample, the response of cells was recorded as a time-series of potentiometric signal (measured in Volts). The measurement period of every measurement was 180 s, sampling rate was 2 Hz resulting on 360 values/sample data collection. At the end of the measurement, the final value of the cells response was uploaded into the cloud server, followed by a calculation process using an algorithm where the result was compared with the data stored arrays, and the result (Below or Above MRL, Test again) appeared on the smartphone screen (Figure 1D).

#### 2.5.2. The Algorithm for Data Acquisition and Signal Processing

Primarily, the purpose was to build data-stored result arrays for Control, Below or Above MRL measurements, in order for the system to classify the samples according to the measurements. Each test produces a time-series consisting of 360 measurements of voltage detection. Based on initial experiments performed with known concentrations of the substance (data not shown), it was observed in just a few cases that the mean values of some time-series significantly differed (80%) from the rest of the data set that normally ranged from 0.024 to 0.167V. These differences were noticed to be either due to a wrong connection of electrode with the device or due to the incorrect addition of biosensors (connection of cell-containing solution drops) on the electrode surface (insufficient cell contact with the electrode surface). In the experiments that followed, the average voltage of each channel time-series was calculated, taking into account only the values which differed from outliers’ values. Afterward, the relative values between pairs of cells were calculated by the following equation:

(1) $$R=\frac{Vero\left(w/Ab\right)}{Vero\;\left(w/o\;Ab\right)}$$

were Vero (*w*/*Ab*) is the response of Vero cells with antibody and Vero (*w*/*o Ab*) is the response of Vero cells without antibody, as shown in the arrangement in Figure 1B.

Then the absolute relative error (ABS) of each value was calculated, and if it exceeded a specific threshold that was set, then the value was rejected and not used for further analysis, while the values below this threshold were used to build the data stored arrays.

After creating the stored arrays, the system was able to sort each test into a category and display to end-user the measurement result. The measurements followed the same processing logic used to build the database. In this case, if only one relative value of the 8X electrode was below the set ABS threshold then the system generated a message alerting the user to repeat the measurement, and if more than one relative value was present, further processing took place. Finally, the system used a paired t-test comparing the relative values with the reference values and the comparison results were displayed on the smartphone application. 

#### 2.5.3. The Software Interface

Building the software interface was a key aspect for system development. The Google Firebase (https://console.firebase.google.com) was used as Backend service to store, authenticate users, secure and calculate the necessary processing. Using the step by step approach of the newly developed algorithm (Section 2.5.2) and the Google cloud functions, we used the following services: (a) Firebase Authentication (b) Firestore DB (c) Google cloud functions to integrate user data with DB (d) REST API to calculate the result (e) Google cloud storage to securely store the raw data per test.

### 2.6. Conventional Sample Analysis

Samples were additionally analyzed using GC/MS at Interuniversity Research Institute for Molecular Recognition and Technological Development (IDM), Polytechnic University of Valencia. The method was validated and accredited according to UNE-EN-ISO/IEC 17025:2017 international standard.

## 3. Results and Discussion

### 3.1. Cells with or without Antibody Response to the Presence of Acetamiprid

The present study developed a biosensor system that utilized Vero cells that were modified in such a way to carry a large number of anti-acetamiprid antibodies to their membrane. Binding of acetamiprid molecules to the corresponding antibodies, in accordance with the proven principle of molecular recognition through membrane engineered would cause alteration of the cell membrane potential [26,30]. As proven in previous studies [24,25,27], the BERA sensor response is directly associated with the hyperpolarization (negative change) or depolarization (positive change) of the cell membrane.

**Figure 2:**
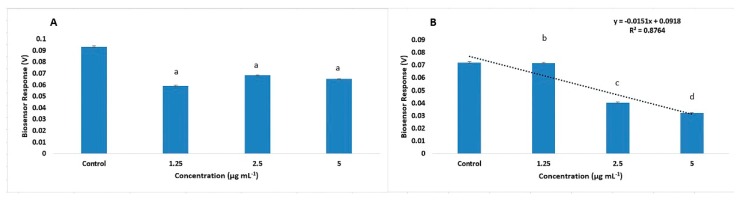
Biosensor response against standard solutions of acetamiprid. Zero acetamiprid concentration was considered as control. Non-engineered Vero cells (i.e., bearing no acetamiprid-specific antibodies) did not show specific responses to the presence of acetamiprid at increasing concentrations (**A**). On the other hand, membrane-engineered cells with antibodies against acetamiprid responded to dilutions of acetamiprid with considerable membrane hyperpolarization (**B**). (Pictured is *n* = 12 replication for each sensor for each different concentration and error bars represent standard errors of the average value of all replications: 768 time-series). Columns with same letters indicate statistically non-different values (*p* < 0.05) and columns marked with different letters indicate significantly different values (*p* < 0.05).

The results of acetamiprid measurements in different dilutions with BERA biosensors are shown in Figure 2. Sensors containing Vero cells modified to carry acetamiprid-specific antibodies to their membrane, responded to dilutions of pure acetamiprid. Results showed considerable membrane hyperpolarization, as indicated by the negative increase in sensor potential with increasing acetamiprid concentrations (Figure 2B). The response among the different acetamiprid concentrations was statistically significant while the distinction between Below MRL (control, 1.25 μg mL^−1^, 2.5 μg mL^−1^) and Above MRL (5 μg mL^−1^) was possible. On the other hand, the non-engineered cells (Figure 2A) seem to react in the presence of acetamiprid but the differences on response was statistically non-significant. At the same time, the response was not a result of a specific reaction and therefore the distinction between Below MRL (control, 1.25 μg mL^−1^, 2.5 μg mL^−1^) and Above MRL (5 μg mL^−1^) was not possible. The observed pattern of membrane-engineered cell response is in agreement with previously published experiments concerning superoxide [27] as well as 2,4,6-trichloroanisole (TCA) [30], indicating that the biosensor operating principle is indeed based on the membrane engineered biorecognition elements.

### 3.2. Biosensor Response to Spiking Lettuce Extract Samples

**Figure 3:**
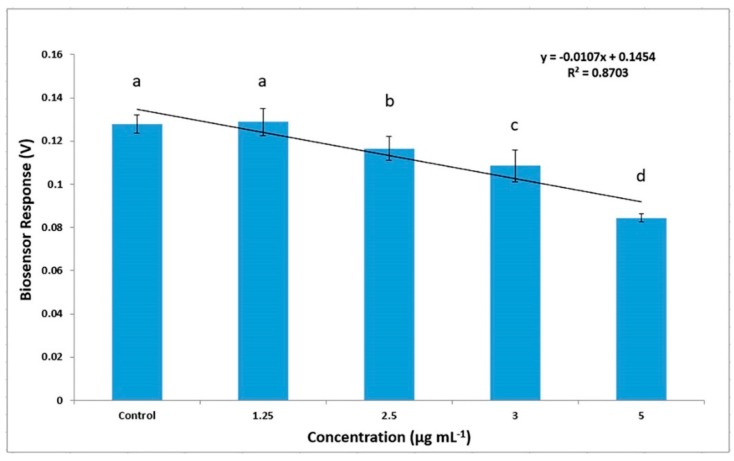
Biosensor response against various concentration of acetamiprid in lettuce extract. Sensor response is expressed as a change in the membrane potential of membrane-engineered cells with antibodies against acetamiprid (*n* = 12 replication each sensor for each different concentration and error bars represent standard errors of the average value of all replications: 480 time-series). Columns with same letters indicate statistically non-different values (*p* < 0.05) and columns marked with different letters indicate significantly different values (*p* < 0.05).

In order to evaluate the feasibility of using the proposed method for routine analysis, the method was further applied for the determination of acetamiprid in lettuce samples. Due to the fact that acetamiprid residues were not detected in the available (market collected) lettuce samples, acetamiprid had been added at different concentrations ranging from 1.25 to 5 μg mL^−1^. The observed results (Figure 3) showed a higher biosensor response (0.43–0.52 mV) compared to the free extract samples (Figure 2B) due to the enhanced matrix effect of the lettuce samples. However, according to two-tailed Student’s T Distribution the response curves of acetamiprid in buffer and lettuce extracts have no significant difference (*p* = 0.94) with an alpha level of 0.05. By calculating the average biosensor response to all the experimental replications, it was concluded that the biosensor was able to detect acetamiprid at all different concentrations, making the system suitable for the detection of acetamiprid in lettuce samples, since it could detect levels below the MRL according to Regulation (EC) No 396/2005 (3 μg mL^−1^). As shown in Figure 3, the detection system responded linearly with decreasing values as the concentration of acetamiprid in the samples increased. The proven linear relationship was *y* = −0.0107*x* + 0.1454 (R^2^ = 0.8703) in lettuce samples with different acetamiprid concentrations. The repeatability of each measurement was tested by (a) analyzing each sample at the same time on all eight different measurement channels and (b) repeating the measurements at three different time periods. The response from each measurement against the acetamiprid calibration standard was quite reproducible (variation 1%–3%). A higher variation was noticed in the determination of acetamiprid in lettuce samples (5%–9%), probably due to the chemical modification of extract composition between the different assay periods.

### 3.3. Database creation

Subsequently, and while it has been previously proven that the detection method works with lettuce extracts, a database has been created in order to give a direct and automated result to the user without requiring any further processing. The results used to create the database were previously processed by the algorithm developed and described in Section 2.5.2. The available data was 972 time-series (each containing 360 measurements). Specifically, 480 time-series were included with Above MRL samples and 492 time-series with Below MRL samples. According to Regulation (EC) No 396/2005 the MRL for acetamiprid for lettuce are 3 μg mL^−1^. For samples considered Above MRL, 9 different acetamiprid concentrations in lettuce were used: 15, 10, 8.75, 7.5, 6.25, 5, 4.5, 4, 3.5 μg mL^−1^, and for the samples considered Below MRL, 5 different acetamiprid concentrations in lettuce were used: 3, 2.5, 2, 1.5, 0.5 μg mL^−1^ along with samples that had no acetamiprid (control).

**Figure 4:**
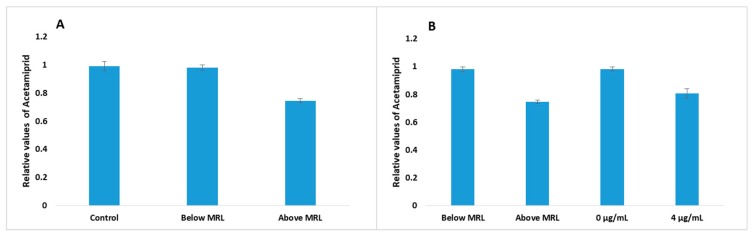
(**A**) Average of relative values from the database after processing with the algorithm. 480 time-series with Above MRL samples and 492 time-series with Below MRL samples and Control samples were used to build the database. (**B**) Comparison of relative values from lettuce extracts with known concentrations of acetamiprid, after processing with the algorithm, with the Above and Below MRL values from the database (128 time-series).

The results that passed the algorithm control were used to build the database. The final values were divided into the following three categories: Above MRL, Below MRL and Control, and for presentation purposes, the average values from the three categories are shown in Figure 4A. However, since it was not possible to differentiate values between Control and Below MRL and since control is considered Below MRL these values were grouped in the same category.

Until confirming the proper functioning of the system, two experiments of eight replicates were performed using lettuce extract with known concentrations of acetamiprid. The average values of the results, after processing with the algorithm, are shown in Figure 4B. The results showed that the lettuce extract at zero concentration (control) of acetamiprid had a similar response to the Below MRL samples, and that the biosensor response to the extract with concentration 4 μg mL^−1^ corresponded to the Above MRL samples. The results obtained from the use of the biosensor against different samples reveal the ability of the system to be used as a screening test for the detection of acetamiprid, and its classification into Above or Below MRL categories.

Based on the above observations a user-friendly interface was created, which, after comparing each test with the Above MRL and Below MRL database, can produce a readable result for the user: ‘Above MRL or Below MRL or Test again’. Test again occurs when the values varies more than 80% from database values.

### 3.4. Biosensor Response and Conventional Analysis of Lettuce Samples Provided from Market

**Figure 5:**
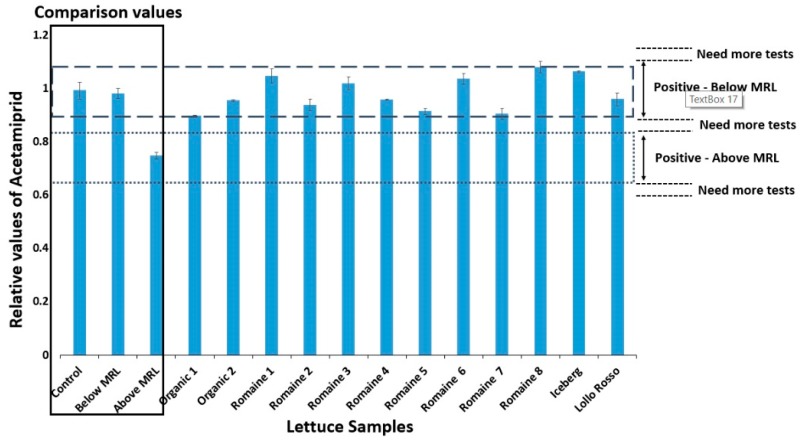
Relative values of lettuce extracts obtaining from a market and analyzed by biosensor system developed. The 96 time-series were obtained from the analysis and results were compared with the average of relative values from the database (columns: control, Below MRL, and Above MRL). The dashed line area corresponds to the Below MRL values and the area of the dots corresponds to the Above MRL values. If the values are at different points than the two areas mentioned above then the experiment has to be repeated.

In order to compare the functionality of the system with a conventional method, 12 lettuce samples were obtained from retail market and extracted according to the protocol described in Section 2.3. The extracts were analyzed by both the conventional method and the newly developed biosensor system. The BERA biosensor response against each sample was initially analyzed by the algorithm and data were uploaded to the database. Samples were then classified into a category and results appeared on the smartphone screen. For convenience, the results extracted from the algorithm are shown in Figure 5. The results indicated that all 12 cases were Below MRL.

The samples were then analyzed by the conventional method (standard based). The results obtained by gas chromatography-mass spectrometry (GC/MS) analysis are presented in Table 1. As can be seen from the conventional analysis the samples contained almost zero concentration of acetamiprid (0.00–0.0129 μg mL^−1^). In accordance with the results from the conventional method, the newly developed approach succeeds in characterizing the samples as below MRL. Hence, the obtained results were consistent with the aim of this study which was to develop a qualitative screening system for acetamiprid detection.

However, despite the possible disadvantages of the novel approach compared to other analytical technologies on offering reliable quantitative results, the present method offers the significant advantage of rapid detection of the contaminated with acetamiprid lettuce, hence providing retailers with the ability to withdraw the products before their distribution to the markets. In addition, the novel assay system is a portable device with an ultra-high-speed response (less than 3 min) compared to other conventional analytical methods [31,32] that require hours and even a few days for detecting acetamiprid. For this reason, the proposed biosensor-based assay can only be considered as a high-speed portable screening system, generating a result that indicates if the sample is Below or Above MRL.

## 4. Conclusions

In conclusion, a simple, rapid, low-cost, and high-throughput portable screening system for acetamiprid has been developed, based on membrane-engineered cells. The novel portable sensor is combined with a sophisticated algorithm embedded in a user-friendly software, obtaining an automated result in a smartphone without the need of examining the biosensor response data by the user. In addition, membrane-engineered cells can be stored for up to four weeks at room temperature without any considerable lose in viability, as already demonstrated in previous studies [27]. An extension of the cell storage time is currently under investigation using different types of cells, both mammalian and non-mammalian in origin. The proposed method has been exploited to detect acetamiprid in real samples and was validated by its good correlation with GC/MS results. Further experiments will investigate the effect of possible interferants, such as other nicotinoids (e.g., imidacloprid) with a similar structure to acetamiprid. Preliminary experiments indicate a strict analyte-specific response for membrane-engineered cells, thus demonstrating the robustness of our approach.

Taking into consideration the results of the present study and the advantages and disadvantages of the novel approach, the cell-based biosensor developed in the present work could be an attractive future technology for field assays, increasing the number of tests done before products reach shelves in stores. The development of a system offering the ability to detect different analytes in the food chain in combination with biosensors’ shelf life augmentation is being currently tested by our research group. 

## Figures and Tables

**Table 1:** Results of lettuce extracts obtaining from a market and analyzed by GC/MS ^a^.

Sample	Concentration (μg mL^−1^) ^b^
LETTUCE 1	0.0002
LETTUCE 2	0.0024
LETTUCE 3	0.0065
LETTUCE 4	0.0129
LETTUCE 5	0.0022
LETTUCE 6	0.0029
LETTUCE 7	0.0027
LETTUCE 8	0.0007
LOLLO ROSSO1	0.001
ICEBERG 1	0.0001
ORGANIC LETTUCE 1	0
ORGANIC LETTUCE 2	0

^a^ Concentrations used for the calibration of the method were 0.0001, 0.001, 0.005, 0.01 and 0.05 μg mL^−1^. ^b^ Concentrations lower than 3 μg mL^−1^ are considered as Below MRL (Regulation (EC) No 396/2005).

## References

[1] Stevens T., Kilmer R. (1999). Pesticide Residues on Fresh Tomatoes and Strawberries. IFAS Ext..

[2] Mestres C., Colonna P., Buleon A. (1988). Characteristics of starch networks within rice flour noodles and mungbean starch vermicelli. J. Food Sci..

[3] US Environmental Protection Agency (2017). Code of Federal Regulations (CFR).

[4] Marın A., Vidal J.M.N., Gonzalez F.E., Frenich A.G., Glass C., Sykes M. (2004). Assessment of potential (inhalation and dermal) and actual exposure to acetamiprid by greenhouse applicators using liquid chromatography–tandem mass spectrometry. J. Chromatogr. B.

[5] Jin D., Xu Q., Yu L., Mao A., Hu X. (2016). A novel sensor for the detection of acetamiprid in vegetables based on its photocatalytic degradation compound. Food Chem..

[6] Yao X.-H., Min H., Lü Z.-H., Yuan H.-P. (2006). Influence of acetamiprid on soil enzymatic activities and respiration. Eur. J. Soil Biol..

[7] Kocaman A., Topaktas M. (2010). Genotoxic effects of a particular mixture of acetamiprid and alpha-cypermethrin on chromosome aberration, sister chromatid exchange, and micronucleus formation in human peripheral blood lymphocytes. Environ. Toxicol..

[8] Kaur R.P., Gupta V., Christopher A.F., Bansal P. (2015). Potential pathways of pesticide action on erectile function—A contributory factor in male infertility. Asia Pac. J. Reprod..

[9] Zhang X., Mobley N., Zhang J., Zheng X., Lu L., Ragin O., Smith C.J. (2010). Analysis of agricultural residues on tea using d-SPE sample preparation with GC-NCI-MS and UHPLC-MS/MS. J. Agric. Food Chem..

[10] Zhang B., Pan X., Venne L., Dunnum S., Mcmurry S.T., Cobb G.P., Anderson T.A. (2008). Development of a method for the determination of 9 currently used cotton pesticides by gas chromatography with electron capture detection. Talanta.

[11] Watanabe E., Miyake S., Baba K., Eun H., Endo S. (2006). Immunoassay for acetamiprid detection: Application to residue analysis and comparison with liquid chromatography. Anal. Bioanal. Chem..

[12] Mateu-Sanchez M., Moreno M., Arrebola F.J., Vidal J.L.M. (2003). Analysis of acetamiprid in vegetables using gas chromatography-tandem mass spectrometry. Anal. Sci..

[13] Wanatabe S., Ito S., Kamata Y., Omoda N., Yamazaki T., Munakata H., Kaneko T., Yuasa Y. (2001). Development of competitive enzyme-linked immunosorbent assays (ELISAs) based on monoclonal antibodies for chloronicotinoid insecticides imidacloprid and acetamiprid. Anal. Chim. Acta.

[14] Hassani S., Momtaz S., Vakhshiteh F., Maghsoudi A.S., Ganjali M.R., Norouzi P., Abdollahi M. (2017). Biosensors and their applications in detection of organophosphorus pesticides in the environment. Arch. Toxicol..

[15] Rodriguez-Mozaz S., Lopez de Alda M.J., Barceló D. (2006). Biosensors as useful tools for environmental analysis and monitoring. Anal. Bioanal. Chem..

[16] Scognamiglio V., Pezzotti G., Pezzotti I., Cano J., Buonasera K., Giannini D., Giardi M.T. (2010). Biosensors for effective environmental and agrifood protection and commercialization: From research to market. Microchim. Acta.

[17] Luque de Castro M.D., Herrera M.C. (2003). Enzyme Inhibition Based Biosensors and Biosensing Systems: Questionable Analytical Devices. Biosens. Bioelectron..

[18] Jaffrezic-Renault N. (2001). New Trends in Biosensors for Organophosphorus Pesticides. Sensors.

[19] Grieshaber D., MacKenzie R., Vöros J., Reimhult E. (2008). Electrochemical Biosensors—Sensor Principles and Architectures. Sensors.

[20] Ronkainen N.J., Halsall H.B., Heineman W.R. (2010). Electrochemical biosensors. Chem. Soc. Rev..

[21] Jiang X., Li D., Xu X., Ying Y., Li Y., Ye Z., Wang J. (2008). Immunosensors for Detection of Pesticides Residues. Biosens. Bioelectron..

[22] Suri C.R., Boro R., Nangia Y., Gandhi S., Sharma P., Wangoo N., Rajesh K., Shekhawat G.S. (2009). Immunoanalytical Techniques for Analyzing Pesticides in the Environment. Trends Anal. Chem..

[23] Kintzios S. (2007). Cell-based sensors in clinical chemistry. Mini-Rev. Clin. Chem..

[24] Mavrikou S., Flampouri K., Moschopoulou G., Mangana O., Michaelides A., Kintzios S. (2008). Assessment of organophosphate and carbamate pesticide residues in cigarette tobacco with a novel cell biosensor. Sensors.

[25] Flampouri K., Mavrikou S., Kintzios S., Miliadis G., Aplada-Sarlis P. (2010). Development and validation of a cellular biosensor detecting pesticide residues in tomatoes. Talanta.

[26] Moschopoulou G., Vitsa K., Bem F., Vassilakos N., Perdikaris A., Blouhos P., Yialouris C., Frossiniotis D., Anthopoulos I., Maggana O. (2008). Engineering of the membrane of fibroblast cells with virus-specific antibodies: A novel biosensor tool for virus detection. Biosens. Bioelectron..

[27] Moschopoulou G., Kintzios S. (2006). Application of membrane-engineering to bioelectric recognition cell sensors for the detection of picomole concentrations of superoxide radical: A novel biosensor principle. Anal. Chim. Acta.

[28] Zeira M., Tosi P.F., Mouneimne Y., Lazarte J., Sneed L., Volsky D.L., Nikolau C. (1991). Full-length CD4 electroinserted in the erythrocyte membrane as a long-lived inhibitor of infection by human immunodeficiency virus. Proc. Natl. Acad. Sci. USA.

[29] Anastassiades M., Lehotay S.J. (2003). Fast and easy multiresidue methods employing acetonitrile extraction/partitioning and dispersive solid-phase extraction for the determination of pesticide residues in produce. J. AOAC Int..

[30] Apostolou T., Pascual N., Marco M.P., Moschos A., Petropoulos A., Kaltsas G., Kintzios S. (2014). Extraction-less, rapid assay for the direct detection of 2,4,6-trichloroanisole (TCA) in cork samples. Talanta.

[31] Dujaković N., Grujić S., Radišić M., Vasiljević T., Laušević M. (2010). Determination of pesticides in surface and ground waters by liquid chromatography–electrospray–tandem mass spectrometry. Anal. Chim. Acta.

[32] Tanner G., Czerwenka C. (2011). LC-MS/MS analysis of neonicotinoid insecticides in honey: Methodology and residue findings in Austrian honeys. J. Agric. Food Chem..

